# Elucidating the Lithiation Process in Fe_3−δ_O_4_ Nanoparticles by Correlating Magnetic and Structural Properties

**DOI:** 10.1021/acsami.3c18334

**Published:** 2024-03-13

**Authors:** Seda Ulusoy, Mikhail Feygenson, Thomas Thersleff, Toni Uusimaeki, Mario Valvo, Alejandro G. Roca, Josep Nogués, Peter Svedlindh, German Salazar-Alvarez

**Affiliations:** † Department Materials Science and Engineering, Uppsala University, P.O. Box 35, 751 03 Uppsala, Sweden; ‡ European Spallation Source ERIC, SE-22100 Lund, Sweden; § Jülich Centre for Neutron Science (JCNS-1) Forschungszentrum Jülich, D-52425 Jülich, Germany; ∥ Department Materials and Environmental Chemistry, Stockholm University, 106 91 Stockholm, Sweden; ⊥ Department Chemistry, Uppsala University, 752 37 Uppsala, Sweden; # Catalan Institute of Nanoscience and Nanotechnology (ICN2), CSIC and BIST, Campus UAB, Bellaterra 08193, Barcelona, Spain; ¶ ICREA, Pg. Lluís Companys 23, Barcelona 08010, Spain

**Keywords:** iron oxide, lithiation, structural transformation, diffraction, magnetism

## Abstract

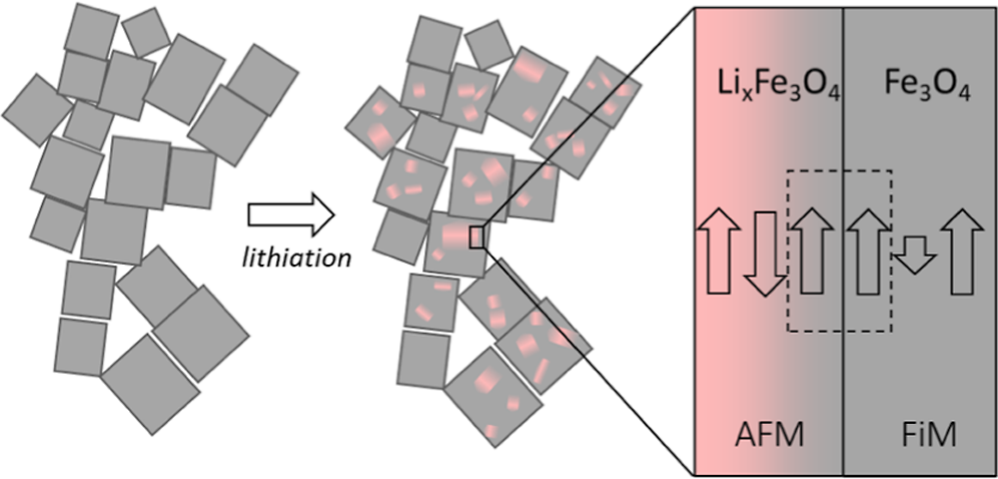

Due to their high potential energy storage, magnetite (Fe_3_O_4_) nanoparticles have become appealing as anode materials in lithium-ion batteries. However, the details of the lithiation process are still not completely understood. Here, we investigate chemical lithiation in 70 nm cubic-shaped magnetite nanoparticles with varying degrees of lithiation, *x* = 0, 0.5, 1, and 1.5. The induced changes in the structural and magnetic properties were investigated using X-ray techniques along with electron microscopy and magnetic measurements. The results indicate that a structural transformation from spinel to rock salt phase occurs above a critical limit for the lithium concentration (*x*_c_), which is determined to be between 0.5< *x*_c_ ≤ 1 for Fe_3−δ_O_4_. Diffraction and magnetization measurements clearly show the formation of the antiferromagnetic LiFeO_2_ phase. Upon lithiation, magnetization measurements reveal an exchange bias in the hysteresis loops with an asymmetry, which can be attributed to the formation of mosaic-like LiFeO_2_ subdomains. The combined characterization techniques enabled us to unambiguously identify the phases and their distributions involved in the lithiation process. Correlating magnetic and structural properties opens the path to increasing the understanding of the processes involved in a variety of nonmagnetic applications of magnetic materials.

## 1 Introduction

Spinel iron oxide nanoparticles have received significant attention due to their interesting magnetic properties, coupled with the large natural abundance of their components and ease of preparation.^1^ Among these, magnetite (Fe_3_O_4_) has been explored in various fields ranging from contrast agents for magnetic resonance imaging, sensing, targeted drug delivery, and energy storage.^2^ The spinel structure is built on an *fcc* array of oxide anions that provides a large number of available octahedral and tetrahedral interstitial sites in the crystal structure. This gives the possibility of hosting additional ions, such as Na^+^ or Li^+^, that can be inserted chemically or electrochemically.^3−13^ In particular, the [B_2_]O_4_ framework of the spinel structure has obtained significant attention due to its potential use as anode, cathode, or electrolyte in secondary Li–ion batteries.^14^

Fe_3_O_4_ possesses a high theoretical storage capacity (925 mA h/g), though the dense spinel structure is reported to be a limiting factor for Li–ion diffusion.^15^ Nanosizing is one strategy to overcome this barrier, as it can shorten the effective diffusion pathways of Li–ions and lead to improved electrochemical performance up to some extent. Previous studies show that below a certain particle size (≲50 nm), the increased surface area of the particles combined with reactions with the electrolyte cause a faster degradation of the electrode.^16,17^ Regardless of the particle size, the lithiation of Fe_3_O_4_ is described as a two-step reaction in which insertion and conversion reactions occur in a sequential manner.^18^ Fe_3_O_4_ first undergoes an insertion reaction resulting in Li_*x*_Fe_3_O_4_ (0 < *x* < 2), where the lithiation forces a change in the valence state of iron and also causes a structural transformation from spinel to a rock salt-like (Li_*x*_FeO_4_) phase.^19^ This happens via a topotactic redistribution of cations from tetrahedral to octahedral sites while maintaining the oxygen *fcc* lattice.^14^ Beyond a critical lithium concentration (*x* ≳ 2), the reduction of iron to metallic iron results in the segregation of Li_2_O.

Additionally, Fe_3_O_4_ was explored as a model magnetic system. Interestingly, the magnetic properties in the Fe_*x*_O_*y*_ system depend critically on the oxidation state of the Fe ion and the doping. In this framework, the structural changes induced during the lithiation of Fe_3_O_4_ are accompanied by changes in its magnetic behavior, spanning from ferrimagnetic Fe_3_O_4_ to an antiferromagnetic LiFeO_2_^20,21^ to ferromagnetic Fe. Although the correlation between the structural and magnetic properties during chemical transformations, e.g. ref (22), could shed light on diverse aspects of the process, it has been seldom explored to study the lithiation process.^9,23^

In this work, we have investigated the chemical lithiation of 70 nm cubic shaped iron oxide nanoparticles from structural and magnetic viewpoints by using X-ray diffraction (XRD), total X-ray scattering with pair distribution function (PDF) analysis, scanning transmission electron microscopy (STEM), and magnetization measurements. The results evidence that the combined structural-magnetic approach gives additional insights into the lithiation process, which is challenging to obtain from a single type of characterization.

## 2 Methods

### 2.1 Synthesis of Cubic Fe_3_O_4_ Nanocrystals

Fe_3_O_4_ particles with cubic shape were synthesized by decomposition of Fe(acac)_3_ (97%, Sigma-Aldrich) in the presence of oleic acid (OA) (99%, TCI) and sodium oleate (99%, TCI), as reported in refs (24 and 25). 6 mmol of Fe(acac)_3_ was mixed with 12 mmol OA and 1 mmol Na-oleate by adding 40 mL dibenzyl ether (98%, Sigma-Aldrich), 40 mL 1-octadecene (90%, Sigma-Aldrich), and 12 mL tetradecene (92%, Sigma-Aldrich) solvents. The mixture was magnetically stirred and degassed at room temperature for 1 h, followed by the mixture being heated to reflux temperature (∼290 °C) at a rate of 20 °C/min under a nitrogen atmosphere for 30 min before cooling to room temperature. The particles were washed 3–4 times with a mixture of toluene and ethanol (1:4) and centrifuged at 6000 rpm for 5 min, discarding the supernatant. This step was repeated until the organic amount was less than 2%. The obtained Fe_3_O_4_ nanoparticles with cubic shape morphology (edge length of *l* = 73 ± 9 nm) (see Figure S1) were used as starting powders for the chemical lithiation procedure.

### 2.2 Chemical Lithiation of Fe_3_O_4_ Nanoparticles

Approximately 20 mg of Fe_3_O_4_ nanoparticles were added to a 1.6 M *n*-butyllithium (BuLi) anhydrous hexane solution to obtain various nominal molar ratios of *x* = Li/Fe_3_O_4_ = 0, 0.5, 1, and 1.5. The mixtures were left to react for 3 days at room temperature under magnetic stirring in an argon-filled glovebox environment, where the O_2_ and H_2_O levels were kept below 1 ppm. After that, the remaining liquid was discarded, and the particles were washed in hexane a couple of times to remove any excess BuLi with the help of an external magnet to hasten the sedimentation rate of the particles. The particles were then dried to obtain powders for further analysis.

### 2.3 Inductively Coupled Plasma-Optical Emission Spectrometry

Inductively coupled plasma-optical emission spectrometry (ICP–OES) was used to estimate the Li and Fe amounts in the sample. The lithiated powders were digested in 0.3 mL of 37 wt % hydrochloric acid (Sigma-Aldrich) for several hours and then further diluted with 5 wt % nitric acid (Fisher Scientific). Before the measurement, the samples were filtered with 0.45 μm syringe filters (Whatman). The Avio 200 Scott/Cross-Flow configuration was used for ICP measurements. A calibration curve was formed for the measurements using the multielement standard Li and Fe (CPAchem). Concentrations of 0, 0.1, 0.5, and 1 ppm for Li as well as 0, 1, 10, and 50 ppm of Fe were used to create a 4-point linear regression. All measured values are within a relative standard deviation of 2%. The experimental molar ratios obtained using ICP–OES are *x*_exp_ = Li/Fe_3_O_4_ = 0, 0.3444(8), 0.7839(8), and 1.200(2), which correspond to the nominal values *x* = 0, 0.5, 1, and 1.5, respectively.

### 2.4 Transmission Electron Microscopy

The lithiated Fe_3_O_4_ nanoparticle powders were dispersed in toluene and drop-cast on carbon-coated grids (Ted Pella, Cu 400 mesh) to perform TEM measurements using a Titan Themis microscope (FEI) using a Schottky field-emission gun operating at an accelerating voltage of 200 kV.

### 2.5 Scanning Transmission Electron Microscopy and Electron Energy Loss Spectra

The 4D-STEM images were acquired using the custom-made software St4DeM,^26^ which utilizes Digital Micrograph’s SDK environment for fast software-synchronized scanning. Micrographs of two samples were recorded using a Thermo Fisher 300 kV Cs-corrected Themis TEM coupled with a Gatan Oneview camera. A sample holder for air-sensitive specimens (Thermo Fisher) was used for the lithiated sample to avoid oxidation. During the Microbeam STEM mode at 57k× magnification, a condenser aperture of 50 μm, a spot size of 7, an indicated camera length of 145 mm, 10 ms of exposure time, a measured screen current of 0.046 nA, and an indicated convergence angle of 0.21 mrad were used to collect the data.

The spectrum images (Supporting Information) for electron energy loss spectra (EELS) data were recorded using a GIF Quantum ER with an indicated convergence angle of 1.04 mrad and a collection angle of 11.49 mrad. The spectra were acquired with a dispersion of 0.25 eV/channel. The analysis of the spectra is studied using Digital Micrograph software.^27^ Initially, the spectra are aligned with respect to the zero-loss peak, and a power-law fit is selected as the background model for their background correction.

### 2.6 XRD and Total Scattering

Transmission XRD measurements were performed on epoxy-sealed glass capillaries to prevent oxidation and hydrolysis when in contact with air, using a D8 Advance single crystal diffractometer (Bruker) with Mo Kα radiation (λ = 0.71073 Å) and a 2D Photon 100 detector (1024 × 1024 px^2^, pixel size = 96 × 96 μm^2^) using an exposure time of 120 s. The raw detector images were reduced into 1D data using the pyFAI package.^28^ The Rietveld refinement of the structures was performed with TOPAS Academic (V6) software. The peak profiles were described by separate Gaussian and Lorentzian components. The instrumental contribution to the peak shape was corrected by performing Pawley refinement on an NIST Si standard reference material, and its contribution is included in each refinement. The total X-ray scattering data were collected at beamline P21.1 at DESY with an X-ray wavelength of 0.122 Å using a 4096 × 4096 px^2^ 2D detector (PerkinElmer). Azimuthal integration and calibrations were performed using the pyFAI package. PDFs were generated with *Q*_max_ = 25 Å^–1^, *Q*_max,inst_ = 26 Å^–1^, *Q*_min_ = 0.1 Å^–1^, and *r*_poly_ = 0.9 using the xPDFsuite.^29^ The PDF data fits were evaluated for a low *r*-range between 1 and 25 Å by using the PDFgui program.^30^ The Rietveld refinements were used as starting models for the PDF fits, where the scale factor, lattice constant, linear atomic correlation factor (delta2), isotropic thermal parameters (*u*_11_, *u*_22_, and *u*_33_), occupancy, and symmetry constraints were refined in consecutive order.

### 2.7 Raman Spectroscopy

Raman measurements were conducted at room temperature by means of a Renishaw (inVia) spectrometer with a built-in optical microscope (Leica) and employing, for this purpose, a 532 nm laser excitation wavelength. The latter was provided by a solid-state laser diode (Renishaw, max power of 500 mW). Prior to the sample analyses, a calibration of the instrument was performed through a Si wafer specimen to make sure that a characteristic Raman peak, taken here as a reference, was detected at ∼520 cm^–1^. The Fe_3_O_4_ powder was placed onto a glass slide, and a 50× lens was utilized to focus the laser beam onto the sample surface. A low operational laser power was employed (i.e., 0.1% of its maximum nominal value) in order to effectively prevent local heating, which can result in oxidation of the sample. The spectral acquisition time was chosen as 30 s with a series of 70 consecutive accumulations to enhance the signal-to-noise (S/N) ratio in the ultimate spectra. Minimization of laser beam exposure for the sample in-between subsequent accumulations was applied to prevent possible sample degradation during data collection.

### 2.8 Magnetic Measurements

The magnetic properties were measured with a magnetic property measurement system, MPMS-XL (Quantum Design). The temperature-dependent DC magnetization curves were collected in field-cooled (FC) and zero-field-cooled (ZFC) modes from 300 to 10 K in an applied magnetic field of 0.01 T. Prior to the measurements, the magnetic field was quenched to less than 0.4 mT before starting the ZFC procedure. Exchange bias measurements were recorded at 10 K for each sample, cooling from 300 K under an applied field of 5 T. The corresponding exchange bias values were calculated as using equation μ_0_*H*_E_ = μ_0_(|*H*^−^| − |*H*^+^|)/2, where *H*^+^ and *H*^–^ are the coercive fields for the negative and positive field axes, respectively.

## 3 Results and Discussion

### 3.1 Structural and Morphological Changes upon Lithiation

**Figure 1:**
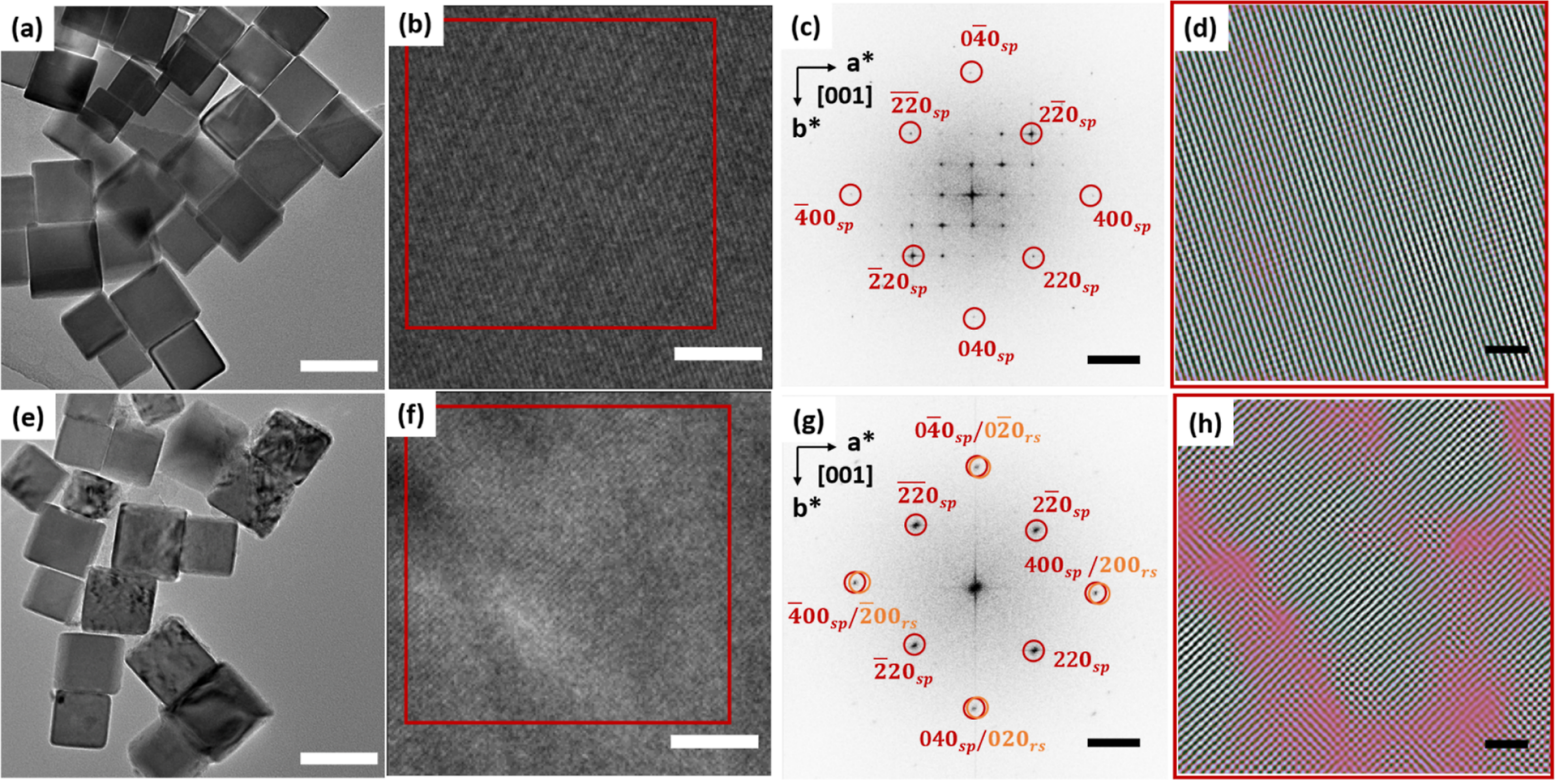
TEM images of the pristine (*x* = 0) and lithiated (*x* = 1.5) nanocubes. (a,e), HRTEM images at higher magnifications of both nanoparticles (b,f) with corresponding FFT analysis on the right side (c,g), and inverse FFT of 220 spinel reflections for each sample (*x* = 0 and *x* = 1.5) from the red box area (d,h). The spinel and rock-salt diffraction spots are denoted as sp and rs, respectively. Scale bars in (a,e) = 100 nm, (b,f) = 5 nm, (c,g) = 2 nm^–1^, and (d,h) = 2 nm, respectively.

Figure 1 shows TEM images and an image analysis of the particles before (*x* = 0) and after lithiation (*x* = 1.5). Figure 1b,f are high-resolution TEM (HRTEM) images of a selected particle from Figure 1a,e, respectively. The contrast variation in Figure 1e clearly shows that the internal structure of the particles changes upon lithiation (*x* = 1.5) while maintaining the particle size and shape (see Figure S1 for the size determination details). The fast-Fourier transform (FFT) analysis of the HRTEM images from Figure 1b,f is presented in Figure 1c,g, respectively. The diffraction spots are indexed using a spinel structure with space group *P*4_3_32, which contains superstructural reflections (not indexed) from the ordering of vacant sites in maghemite (γ-Fe_2_O_3_)^31^ structure, as confirmed by Raman measurements as well (see Figure S2).^32^Figure 1g shows that the superstructural reflections vanish after lithiation, indicating that the vacant sites are being filled, likely by Li (or Fe) ions. Additionally, at this level of lithiation (*x* = 1.5), structural transformation from the spinel to the rock salt phase is expected to take place.^4,33^ The transformation to rock salt structure is likely to be topotactical,^18^ whereby many reflections of both the spinel and rock salt structures overlap, except for the 220 reflections (see indexing in Figure 1g). By using the 220 spinel-only reflections from marked square regions in Figure 1b,f, the inverse FFT (iFFT) images in Figure 1d,h are obtained, respectively. The nonmonotonic contrast variation in Figure 1h from lithiated particles is probably due to the formation of smaller rock salt crystallites, generating dislocations at the spinel-rock salt interface. However, thickness variations cannot be discarded either. Besides, complementary iFFT images for both pristine and lithiated samples using the 400_sp/rs_ diffraction spots are also depicted in Figure S3 to evaluate the change with lithiation. Although both spinel and rock salt diffraction spots overlap for the (400) plane, the lithiated sample shows a more nonmonotonic contrast variation than the pristine particle, which indicates the presence of dislocations along the (400) crystallographic planes as well.

The results from the electron microscopy analysis suggest that, upon lithiation (*x* = 1.5), there is a chemical and topotactical transformation from spinel to rock-salt structure where the particle size and shape are more or less preserved. Surprisingly, the lithiated particles do not show a clear core–shell morphology but rather a mosaic-like morphology with a significant amount of disorder at the spinel-rock salt interface.

**Figure 2:**
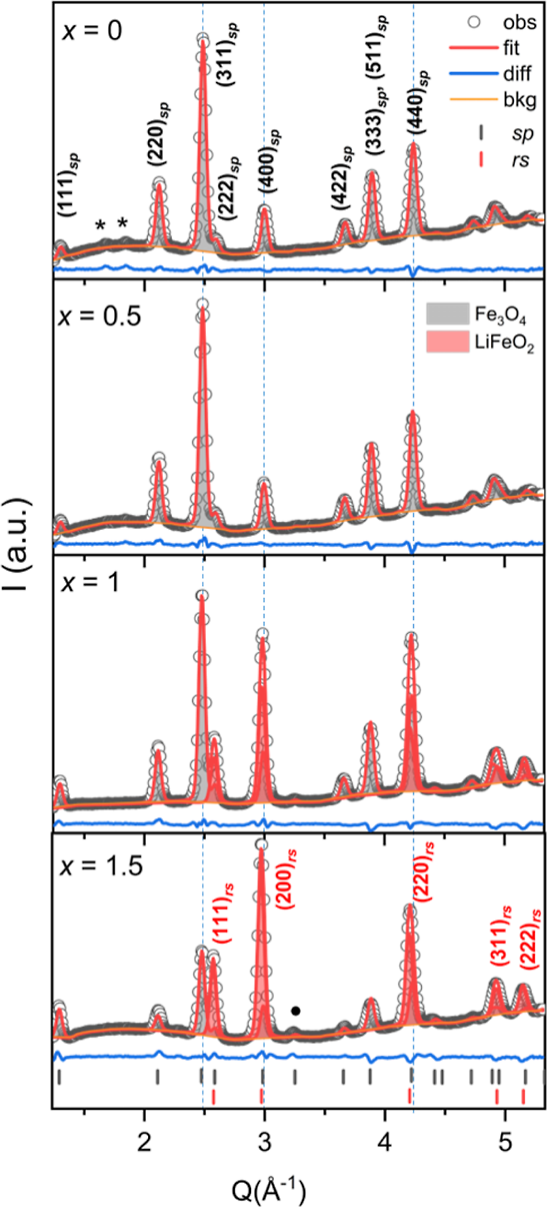
XRD data and Rietveld refinements of particles at different degrees of lithiation. The two main structures, Fe_3_O_4_ (inverse spinel, denoted as sp) and LiFeO_2_ (rock-salt, denoted as rs), are indicated at each degree of lithiation (0 ≤ *x* ≤ 1.5). The black symbols correspond to observed data points, the red curve to the Rietveld fit, and the blue line to the difference between the experimental data and the fit. The bars at the bottom indicate the peak positions for both phases. The stars indicate the position of the superstructure reflections of the maghemite phase. An additional peak at ca. 3.2 Å^–1^ (black dot) can be related to metallic iron. The diffraction (*hkl*) planes of each phase are shown above the peaks.

Further structural analysis was carried out using powder XRD, and the results can be seen in Figure 2. The obtained lattice parameters and phase composition analysis are shown in Figure S4, whereas all the refined parameters, such as occupancies, atomic positions, bond angles, and bond lengths for each degree of lithiation, are summarized in Table S1. The most remarkable changes in Figure 2 are the variation in peak intensity ratios along with the peak shifts and the disappearance of superstructural reflections [marked with asterisks (*)] at low *q*-range, while going from the pristine state (*x* = 0) to the lithiated state (*x* = 1.5). For the pristine sample (*x* = 0), its lattice parameter is determined to be ca. 8.3857(9) Å, which suggests that the overall structural composition is closer to magnetite (*a* = 8.396 Å) rather than maghemite (*a* = 8.340 Å),^34,35^ despite the XRD pattern and the FFT in Figure 1c showing extra vacant site reflections attributed to maghemite. This is most likely due to the ease of oxidation of Fe_3_O_4_ in air,^36^ resulting in an iron-deficient surface layer of the particle with a composition close to γ-Fe_2_O_3_. Previous reports have indeed shown that air-exposed magnetite nanoparticles consist of maghemite, resulting in a graded core–shell configuration with an iron–oxygen gradient along the radius of the particle.^36,37^ Thus, it is expected that the surface layer consists of the maghemite phase for the pristine particle, which is the origin of the superstructural reflections.

As lithiation increases up to *x* = 0.5, the formation of the rock salt structure is expected. However, other structures, apart from the inverse spinel, are not observed in the refined XRD pattern. There is only a minor change in the superstructural reflections at the low *q*-range, which diminish compared to the pristine state. A theoretical investigation of the Li–Fe–O system suggests that lithiation at *x* = 0.5 can hinder the formation of rock salt structures in the presence of vacancies, such as those in maghemite.^33^ Thus, the incorporation of Li in the γ-Fe_2_O_3_ layer would explain both the vanishing superstructure peaks and the absence of a rock salt phase for *x* = 0.5. There is only a slightly increased occupancy of the octahedral (16c) site from 0.938(7) to 0.957(8), while the tetrahedral (8a) site occupancy remains constant. Indeed, previous studies reported that Li (or Fe) ions occupy initially the empty interstitial octahedral 16c sites owing to the electrostatic stability of Li-ion with the neighboring Fe_8a_^III^ ions.^4,33^ However, beyond the critical amount of Li (*x*_c_) ions at the octahedral sites, repulsive electrostatic forces between the Li_16c_^I^ and Fe_8a_^III^ increase and displace Fe_8a_^III^ ions to adjacent 16c sites, starting the transformation from spinel to rock salt structure. Thus, reaction up to nominal *x* = 0.5 can be expressed as *x*_c_ with the following eq 1, considering that the spinel structure is maintained
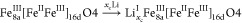
1

As further lithium was added up to *x* = 1, the rock salt structure emerged, approaching 43% with a lattice parameter *a*_rs_ = 4.2179(5) Å, and the inverse spinel structure showed the lattice parameter *a*_sp_ = 8.4066(9) Å. This represents practically no significant change for the spinel structure and 1.4% unit cell expansion for the rock salt structure compared to their reported bulk values,  Å,^34^ and *a*_LiFeO_2__ = 4.16 Å,^38^ respectively. The occupancy of Fe at (8*a*) tetrahedral sites decreased from 1.00(1) to 0.92(3) while the occupancy of Fe at (16c) octahedral sites increased from 0.95(7) to 1.00(3), when going from *x* = 0.5 to *x* = 1. The occupancy values suggest that Fe-ions at tetrahedral sites move to octahedral sites beyond a critical concentration of Li (0.5 < *x*_c_ ≤ 1), which can be expressed by the following equation (eq 2)
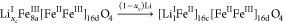
2

As we proceed with one more lithiation step to *x* = 1.5, there is a gradual increase in the rock salt phase contribution to 67% with a lattice parameter *a*_rs_ = 4.2279(6) Å and inverse spinel structure showing a lattice parameter *a*_sp_ = 8.400(1) Å, evidencing almost no change in the lattice parameter of the spinel structure, while the rock salt structure exhibits 1.6% unit cell expansion compared to the bulk value. On the other hand, the occupancy of Fe at the tetrahedral (8a) sites decreased even further to 0.67, while the octahedral (16c) sites are fully occupied, which implies that a more prominent structural transformation from spinel to rock salt phase occurs. The results further confirm the phase contrast in the HRTEM image from *x* = 1.5 that is associated with rock salt phase formation and disorder at the spinel-rock salt interface. A small additional peak at ca. 3.2 Å^–1^ that can be related to metallic iron with a compressed lattice parameter of *a*_bcc_ ≈ 2.78 Å is observed. This could arise from reduced iron species at the surface of the particles. Earlier articles indeed described the extrusion of metallic Fe at all stages of lithiation, but mainly at high temperatures.^7,39^

Here, based on the XRD analysis, we can conclude that Li-ions initially occupy the empty interstitial sites up to a critical concentration, and the spinel structure is maintained with a slight expansion of the unit cell. Above the critical concentration of Li (*x*_c_), a structural transformation from spinel to rock salt structure takes place.^4,19^ In fact, by comparing the results for the *x* = 0.5 and *x* = 1 lithiated samples, it is possible to determine that the critical concentration, *x*_c_ lies in the range 0.5 < *x*_c_ ≤ 1 for iron-deficient magnetite (Fe_3−δ_O_4_) as the starting composition. This is the first detailed experimental validation for this interval of critical concentration values to initiate phase conversion in this compound, which can provide valuable information for the Li–Fe–O phase diagram at low lithium concentrations. Besides, a unit cell expansion is observed in both phases, which is possibly due to the mosaic-like distribution of the phases within the particle, in contrast to the core–shell morphology, where usually the shell exerts a compressive force on the core, resulting in lattice contraction/expansion to accommodate the stresses at the interface.^40,41^ Although there is no significant contraction of the lattice parameters of the contributing phases, iFFT images of the lithiated sample indicate that there is strain at the boundary between the two phases as a result of their lattice mismatch. Note that the interfacial strain is likely to contribute to the diffraction peak width; however, the peak widths (Table S2) decrease slightly with lithiation. These two observations can be accommodated simultaneously if there is particle coalescence to a certain extent.

**Figure 3:**
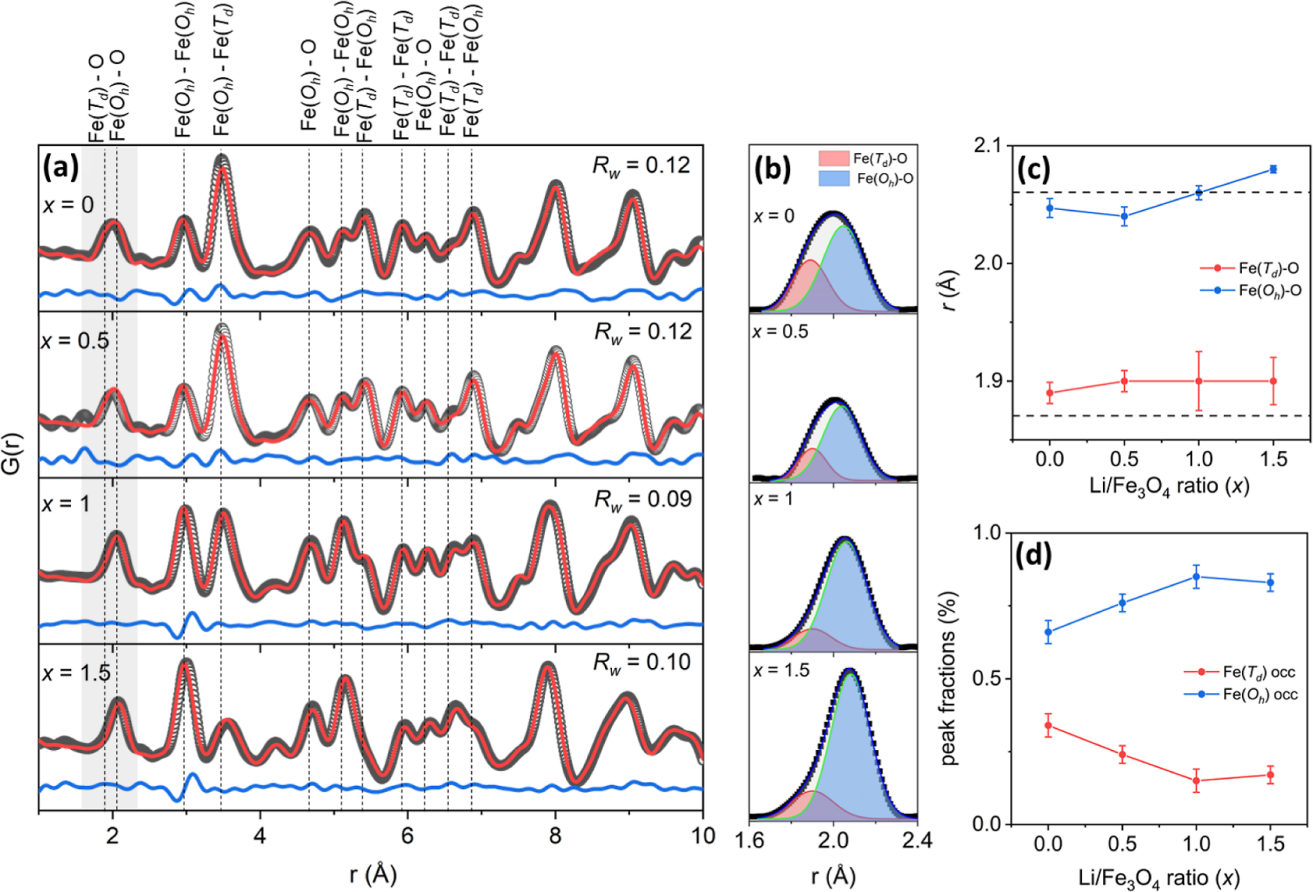
Pair-distribution function analysis at different degrees of lithiation. (a) Open circles, red solid line, and blue bottom line correspond to the experimental data, calculated pattern, and their difference curve, respectively. The assignment of the distances for the different atomic pairs and the corresponding *R*_w_ fit factor are indicated for each degree of lithiation. (b) Gaussian fits for the first peak at 2 Å to deconvolute bond distances of Fe(*T*_*d*_)-O and Fe(*O*_*h*_)-O pairs. Results from the multipeak fit analysis showing (c) bond distances of each pair and (d) peak area ratios to account for the bond-pair population proportional to occupancy of each site, i.e., Fe(8a) and Fe(16d)-sites, respectively. (The dash lines in (c) represent the theoretical bond distance between Fe(*T*_*d*_)-O and Fe(*O*_*h*_)-O for a typical magnetite, as a guide for comparison.).

The changes in the local crystal structure due to the insertion of lithium were investigated by analyzing the total scattering data, and the results are presented in Figure 3. The results from the PDF fitting are also summarized in Table S3 and Figure S4, where volume-weighted phase fractions and calculated lattice parameters are shown together with the XRD analysis results. It is seen that although the values are not identical, the trends in lattice parameters and phase percentage variations are similar for both methods. The PDF fits are displayed in Figure 3a, in which atomic pair distances are shown as dashed lines with corresponding bond pair labels on top of each peak, where *T*_*d*_ and *O*_*h*_ represent tetrahedral and octahedral cation sites, respectively, and the Li ion is not included in the assignments due to its weak X-ray cross-section.

The first emerging peak at ∼2 Å contains both Fe(*T*_*d*_)-O (∼1.87 Å) and Fe(*O*_*h*_)-O (∼2.06 Å) bond distance information,^42^ which shows a shift and a change in peak broadening with the increase in amount of lithiation (see Figure 3 (b)). A multiple peak fit method is used to evaluate Fe(*T*_*d*_)-O and Fe(*O*_*h*_)-O bond distances under the peak at about 2 Å. The initial starting values of both peak centers were chosen from ref (42) and bound within a certain range to prevent overparametrization of the model used for fitting. Figure 3c shows the results obtained from the fit, describing the change in bond distances for Fe(*T*_*d*_)-O and Fe(*O*_*h*_)-O atomic pairs as a function of the Li/Fe_3_O_4_ ratio (*x*) for all of the samples. The Fe(*O*_*h*_)-O and Fe(*T*_*d*_)-O bond distances for a standard magnetite structure are shown as dashed lines at 1.87 and 2.06 Å, respectively (see Figure 3c). For the pristine sample, the Fe(*T*_*d*_)-O bond distance is above the standard value of 1.87 Å, and the Fe(*O*_*h*_)-O bond distance is slightly lower than 2.06 Å. There is also a slight increase in the Fe(*T*_*d*_)-O bond distance and a decrease in the Fe(*O*_*h*_)-O bond distance by lithium insertion at *x* = 0.5, which is a result of stress and local disorder in the structure. As the lithiation proceeds up to *x* = 1, the Fe(*O*_*h*_)-O bond distance increases further, while the Fe(*T*_*d*_)-O bond distance remains almost constant. The clear trend in the Fe(*O*_*h*_)-O bond distance continues for the *x* = 1.5 sample, while the Fe(*T*_*d*_)-O bond distance does not show a significant change in length. The peak area fractions of each bond distance are also evaluated and presented in Figure 3d, where the peak fractions represent the relative population of each bond distance. The peak area fractions are directly correlated to the Fe(*T*_*d*_) and Fe(*O*_*h*_) occupancies, where the peak area fraction of the Fe(*T*_*d*_)-O bond decreases with increasing *x* up to *x* = 1 and seems to saturate at larger *x*-values. On the other hand, the peak area fraction of the Fe(*O*_*h*_)-O bond increases with increasing *x*, supporting the gradual shift of Fe-ions from tetrahedral to octahedral sites upon lithiation, which is in agreement with the results obtained from the Rietveld analysis.

The Fe–Fe bond distances at ∼3 and ∼3.48 Å correspond to Fe(*O*_*h*_)-Fe(*O*_*h*_) and Fe(*O*_*h*_)-Fe(*T*_*d*_), respectively, and show remarkable changes upon lithiation. The most prominent variation is found in the peak intensity ratios, together with minor peak shifts. The peak intensity of the Fe(*O*_*h*_)-Fe(*O*_*h*_) bond distance starts to increase at *x* = 1, where the rock salt structure progressively emerged as a contributing phase. Correspondingly, the Fe(*O*_*h*_)–Fe(*T*_*d*_) bond distance population decreases as the change is caused by the replacement of Fe-ions from tetrahedral to octahedral sites. This structural transformation further confirms the higher fraction of rock salt structure that is formed upon lithiation from *x* = 1 to *x* = 1.5. Phase conversion from spinel to rock salt also induces some local disorder at the boundaries of each structure,^19^ which is evidenced by the Fe(*O*_*h*_)-Fe(*T*_*d*_) bond pair distance increase from 3.48 to 3.55 Å. Similarly, the contrast variation in HRTEM images indicated that there exist dislocations along the (220) plane compared to the pristine sample, where there is no discontinuity in the iFFT image (Figure 1d).

### 3.2 Structure Mapping and Chemical Environment

**Figure 4:**
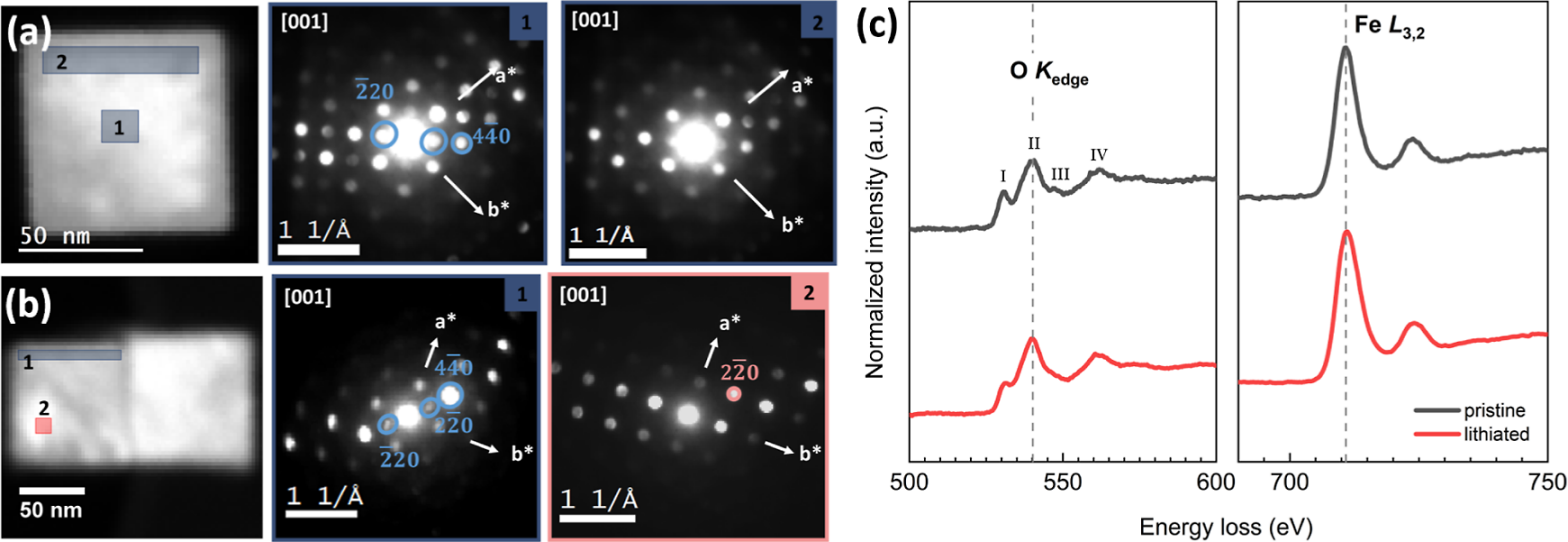
Convergent beam electron diffraction along the chosen regions for pristine (a) and lithiated (*x* = 1.5) (b) nanoparticles. Corresponding diffraction patterns from each representative region denoted as 1 and 2. Blue color represents the spinel phase, whereas the red color indicates the rock-salt phase region and reflections. (c) EELS showing O K-edge and Fe L_2,3_-edge from pristine and lithiated Li_*x*_Fe_3_O_4_ (*x* = 1.5).

4D STEM images from pristine and lithiated particles were collected to determine phase distributions within the particle volume. Two representative regions, one from the center and one from the edge of each particle, were chosen; and are denoted as 1 and 2, respectively, ( Figure 4a,b). The electron diffraction patterns of the pristine particle in Figure 4a show similar reflections for both regions, indicating that the pristine sample consists of a single-crystalline spinel phase. The results for the lithiated (*x* = 1.5) sample shown in Figure 4b reveal that the (220)_sp_ reflection, specific to magnetite, is present in region 1, but it vanishes when scanning from region 1 to region 2, which indicates that only the rock salt structure is present at region 2. This is one of the rare diffraction spots in which there are only reflections of the rock salt structure. Otherwise, both the spinel and rock salt structures coexist, and it is difficult to distinguish the rock salt as its reflections overlap with those of the spinel structure. Despite the challenges in 4D STEM structure mapping, the results point in the same direction as the TEM images that a random mosaic-like phase distribution is possible upon lithiation.

Figure 4c illustrates the oxygen and iron edge spectra for both the pristine and lithiated samples. The oxygen edge spectrum displays four prominent peaks labeled as (I–IV). Notable changes in the spectra upon lithiation include a slight decrease in the intensity of the prepeak at ∼530 eV and the disappearance of a slight bump at ∼548 eV. Besides, the iron edge exhibits no significant changes, possibly due to the low energy resolution of the measurement (>2 eV) and the substantial amount of the pristine state remaining intact even at the highest level of lithiation (*x* = 1.5). In the literature, the prepeak intensity (I) at ∼530 eV is known to be associated with unoccupied 3d states of Fe atoms bonded to oxygen.^44^ The lower intensity of this prepeak in the lithiated sample indicates the presence of a greater number of occupied states arising from hybridized O 2p/Fe 3d bonding, which implies an increased number of Fe^II^ states after lithiation. This finding confirms further the reduction of Fe^III^ to Fe^II^ states upon lithiation.^45^ The presence of a slight bump (III) at ∼548 eV, associated with tetrahedral coordination in the structure, is consistent with the existence of tetrahedral sites in the pristine Fe_3_O_4_ phase,^44^ whereas for the *x* = 1.5 sample, the intensity of this peak vanishes, which can be attributed to the displacement of tetrahedral sites to octahedral sites upon lithiation. In Figure S5, low-loss EELS data from Fe M_2,3_-edge (∼54 eV) and Li K-edges (∼55 eV) are shown for pristine and lithiated samples. However, the peak overlap and limited resolution make it impossible to observe any trends in this energy range.

The combined results obtained from 4D-STEM and EELS suggest a significant change in the coordination number of oxygen, implying variations in the valence state of iron, possibly from Fe^III^ to Fe^II^ state, although there is not much variation at the Fe-edge.^44^ The changes in the prepeak of the O-K_edge_ support the results obtained from structural analysis, such as changes in the Fe–O bond distance, population, and occupancy of Fe cations at different interstitial sites upon lithiation.

So far, despite all the structural information that X-ray and electron microscopy have provided, there is still a lack of information to determine the occupancy and position of Li-ion due to its low X-ray scattering cross-section. Neutron scattering measurements are essential to obtain this information, but they are not always accessible or the amount of sample can be inadequate, which is the case for this study. As an alternative method, magnetism is chosen owing to its sensitivity to structural defects and impurities that can often go beyond the detection limits of XRD.

### 3.3 Correlation between the Structural and Magnetic Properties

**Figure 5:**
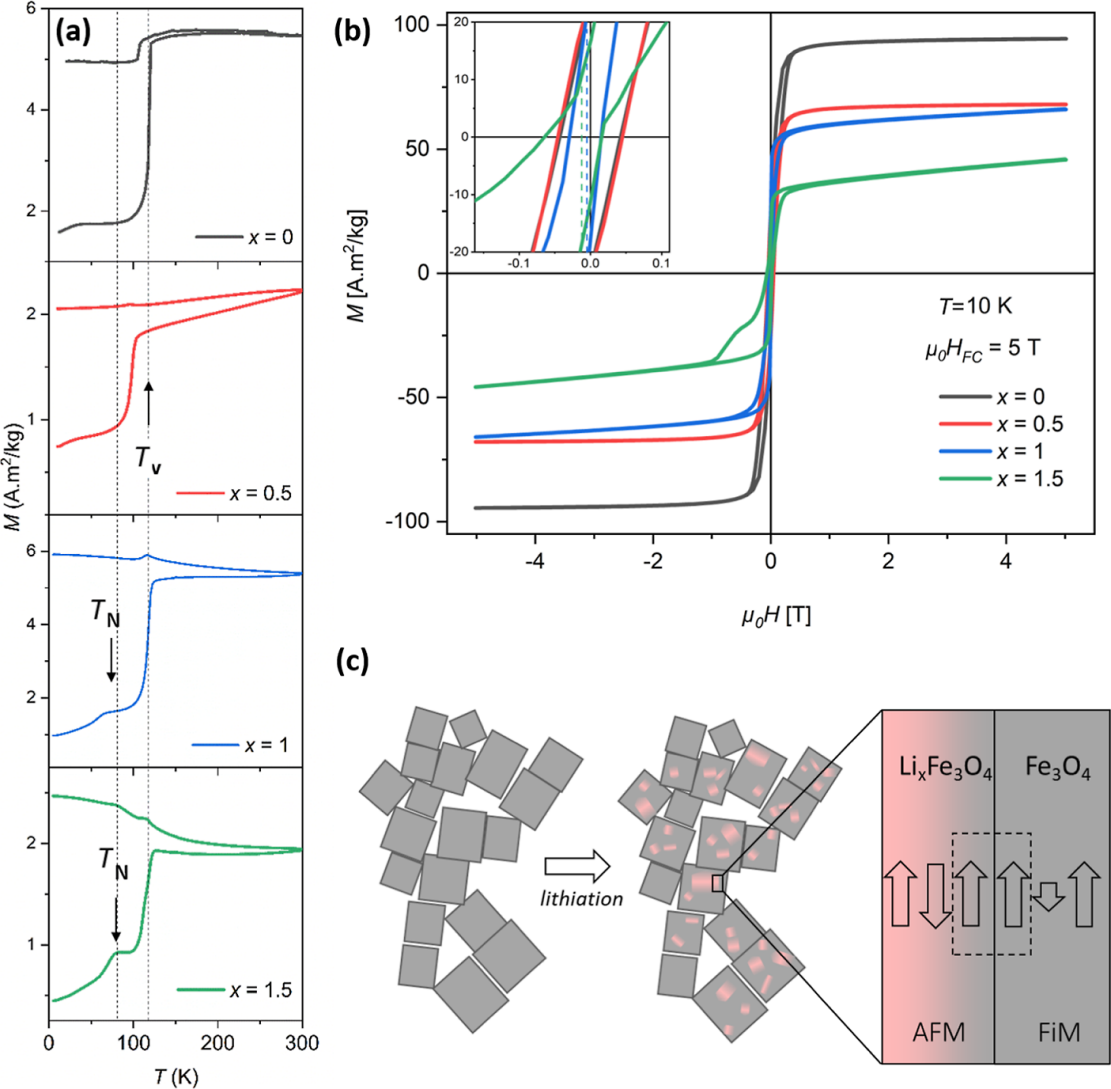
(a) ZFC and FC magnetization versus temperature for the Li_*x*_Fe_3_O_4_ (*x* = 0, 0.5, 1, and 1.5) samples using an applied magnetic field of 0.01 T. The dotted lines represent the Verwey (*T*_v_) and Néel (*T*_N_) transition temperatures of the reported bulk Fe_3_O_4_ and LiFeO_2_ phases, respectively.^38,43^ (b) Hysteresis loops taken at *T* = 10 K after field-cooling (μ_0_*H*_FC_ = 5 T) for the Li_*x*_Fe_3_O_4_ (*x* = 0, 0.5, 1, and 1.5) samples; the inset shows the expanded view of the hysteresis loops at low fields. Schematic diagram of the spin configuration at the AFM-FiM interface (c).

Magnetic measurements were carried out to provide insight into the fine details of the structural changes induced by lithium insertion. First, isothermal hysteresis loops were measured for each sample in the magnetic field range ±5 T at 300 K, as shown in Figure S6. The magnetization values at 5 T, shown in Figure S7, indicate a gradual drop from the pristine (*x* = 0) sample to the lithiated (*x* = 1.5) one. For the pristine sample, the magnetization value at 5 T is ∼89 A m^2^/kg, which is close to the expected saturation magnetization value for the Fe_3_O_4_ phase. The shaded regions in Figure S7 are marked to denote the literature values of *M*_s_ for bulk Fe_3_O_4_ and γ-Fe_2_O_3_ at room temperature as a guide for comparison.^46,47^ Thus, the *M*_s_ values validate the presence of the above-mentioned γ-Fe_2_O_3_ layer on the surface of the pristine sample. For the lithiated (*x* = 0.5) sample, the magnetization value drops to 63 A m^2^/kg and decreases further to 50 A m^2^/kg for *x* = 1, and even further to 27 A m^2^/kg for the *x* = 1.5 sample. The monotonic decrease in *M*_s_ with lithiation corroborates the incorporation of Li in the Fe_3_O_4_ structure and the possible creation of paramagnetic/antiferromagnetic phases, which can also be noticed by the positive slope that develops at the high field region for the lithiated *x* = 1 and *x* = 1.5 samples (see Figure 5b).

Temperature-dependent ZFC and FC magnetization curves under an applied field of 0.01 T were collected to provide further information about possible magnetic phases present in the samples (Figure 5a). The temperature derivative of the ZFC/FC curves is used to determine approximate magnetic phase transition temperatures, as shown in Figure S8. All samples, even the one with the highest degree of lithiation (*x* = 1.5), exhibit a sharp transition at *T* ∼ 120 K, which can be attributed to the Verwey transition temperature (*T*_V_), specific to the magnetite phase.^48−50^ The presence of *T*_V_ in all samples indicates that the magnetite phase remains in the compound after lithiation, which is in accordance with the XRD and PDF results. A significant drop in FC magnetization for *x* = 0 disappears with the increase in lithiation and a remarkable shift (∼20 K) in *T*_V_ is observed for the *x* = 0.5 lithiated sample. This is most likely due to a significant amount of disorder created before the phase transformation takes place.^43,51,52^ For the *x* = 1 and *x* = 1.5 samples, the change in *T*_V_ is subtle, possibly due to stabilization of the structure as a consequence of the completed phase transformation. However, given the high sensitivity of *T*_V_ to the composition of Fe_3_O_4_,^53^ the rather small changes in *T*_V_ may indicate that Li enters the Fe_3_O_4_ structure in an interstitial position rather than substituting an Fe ion. The other distinct feature in Figure S8 is the small kink below 90 K for the *x* = 1 and *x* = 1.5 samples, which is likely to originate from the formation of the antiferromagnetic (AFM) LiFeO_2_ phase. In the literature, bulk LiFeO_2_ is reported to show a Néel temperature (*T*_N_) around ∼90 K.^20,38^ The *T*_N_ values for the *x* = 1 and *x* = 1.5 samples are ∼58 and ∼70 K, respectively, where the deviation from the bulk value can be attributed to expansion of the unit cell parameter and enlargement of the Fe–O–Fe bond distances, although a size effect can also be present. On the other hand, the presence of the AFM phase is not reflected in the FC curve with the increase in lithiation, which could be due to strong magnetic signal from ferromagnetically coupled spins at the interface that will mask the contribution of the AFM phase.

Figure 5b shows the hysteresis loops of all samples at 10 K after field-cooling in a magnetic field of 5 T at from 300 K. The magnetization values at 5 T for the *x* = 1 and 1.5 lithiated samples are reduced compared to those of the pristine sample, and the hysteresis loops exhibit a positive slope in the high field region due to formation of the AFM LiFeO_2_ phase. The inset in Figure 5b indicates a clear shift in the hysteresis loops for these samples along the negative field axis (exchange bias^54^), which can be attributed to interface exchange coupling between the AFM LiFeO_2_ and ferrimagnetic (FiM) Fe_3_O_4_ phases.^54^ According to the electron microscopy results, both phases are randomly distributed in a mosaic-like pattern within the particle (see Figure 5c). The variation of the exchange bias (*H*_E_) values is indicated in Figure S7. There are no significant exchange bias values for the pristine (*x* = 0) and lithiated (*x* = 0.5) samples, whereas *H*_*E*_ sharply increases with increasing lithiation at *x* ≥ 1, likely due to the gradual development of the mosaic-like pattern formed by the random distribution of the AFM and FiM phases.^55,56^ The exchange bias properties in nanostructured disordered systems, represented by the hysteresis loop asymmetry, *H*_E_ and *H*_C_, are a complex combination of several factors, such as the size and magnetic anisotropy of the FiM and AFM phases, the strength of their interface exchange coupling, the degree of pinning of the uncompensated spins in the AFM phase, and the interfacial area between the FiM and AFM phases, among others, that can affect the exchange bias properties^54,57^ Thus, it is difficult to pinpoint the exact origin of the nonmonotonic evolution of *H*_C_ with the increased lithiation (Figure S7). However, given the morphology of the samples, some of the observed effects in the hysteresis loops for the specimens with *x* ≥ 1 could be related to the random crystallographic distribution of the AFM domains.^58,59^

Another feature observed in the hysteresis loops is the presence of an asymmetry for the *x* = 1 and 1.5 samples. The origin of this asymmetry can be attributed to competing anisotropies in the system, which could be due to cationic disorder and anisotropic stress–strain at the interface boundaries.^60,61^ Previous studies have also reported that broken interface exchange bonds and lower crystal symmetry may also result in such asymmetry.^60,61^ Compared to a core–shell nanoparticle with exchange bias, the mosaic-like distribution of AFM|FiM phases creates a dispersion of the FiM and AFM anisotropy axes that compete when the magnetic field direction is reversed, thus yielding higher coercivity with an asymmetry for the case of lithiated samples (*x* = 1 and 1.5).^62,63^ However, other effects, such as a nonhomogeneous distribution of FiM and AMF domain sizes within each particle, could also contribute to the observed asymmetry.

## 4 Conclusions

In summary, we have investigated the correlation between magnetism and structural changes induced by the lithiation of magnetite nanoparticles. It has been observed that the magnetite nanoparticles exhibit a phase conversion from the inverse spinel to the rock salt phase upon chemical lithiation. Good control over the particle size distribution allowed for uniform reactions during the chemical lithiation process. The critical concentration (*x*_c_) of lithium above which Fe_8*a*_^III^ ions are displaced into empty 16c sites is determined to be in the range of 0.5 < *x*_c_ < 1 for Fe_3−δ_O_4_ as the starting composition. Magnetic measurements show that the lithiation results in reduced magnetization and exchange bias behavior when cooled in a magnetic field across the Néel temperature of the AFM LiFeO_2_ phase. The increase in exchange bias values with lithiation is attributed to the formation of mosaic-like LiFeO_2_ subdomains that enlarges the interface area between the AFM (LiFeO_2_)|FiM(Fe_3_O_4_) phases, thus increasing the interfacial exchange coupling. Temperature-dependent magnetization data further supports the AFM behavior of the rock salt LiFeO_2_ phase. This combined structural-magnetic characterization approach could be extended to a broad range of physicochemical applications of magnetic materials to gain insights into the structural changes and a deeper understanding of the chemical processes taking place, potentially also extending to electrochemical measurements. In general, the correlation of magnetic and structural properties allows an increased understanding of the processes involved in a variety of nonmagnetic applications of magnetic materials.

## Supporting Information Available

Additional characterization information on particle size and size distribution, Raman spectra, HRTEM, Rietveld and PDF refinements, EELS, and magnetization curves (PDF)
