# Supplementary material for: Elucidating the Lithiation Process in Fe3−δO4 Nanoparticles by Correlating Magnetic and Structural Properties
Source: ACS Appl Mater Interfaces. 2024 Mar 13;16(12):14799–808. doi: 10.1021/acsami.3c18334 (PMC10982998; doi:10.1021/acsami.3c18334)
Supplement: Supplementary file 1 — am3c18334_si_001.pdf [file am3c18334_si_001.pdf]

SUPPORTING INFORMATION

Elucidating the lithiation process in $Fe_{3-\delta}O_4$ nanoparticles by correlating magnetic and structural properties

Seda Ulusoy,[†] Mikhail Feygenson,[†,‡,¶] Thomas Thersleff,[§] Toni Uusimaeki,[§] Mario Valvo,[‖] Alejandro G. Roca,[⊥] Josep Nogués,[⊥,#] Peter Svedlindh,[†] and German Salazar-Alvarez*,[†]

†*Dept. Materials Science and Engineering, Uppsala University, Box 35, 751 03, Uppsala, Sweden*

‡*European Spallation Source ERIC, SE- 22100 Lund, Sweden*

¶*Jülich Centre for Neutron Science (JCNS-1), Forschungszentrum Jülich, D-52425 Jülich, Germany*

§*Dept. Materials and Environmental Chemistry, Stockholm University, 106 91, Stockholm, Sweden.*

‖*Dept. Chemistry, Uppsala University, 752 37, Uppsala, Sweden.*

⊥*Catalan Institute of Nanoscience and Nanotechnology (ICN2), CSIC and BIST, Campus UAB, Bellaterra, 08193 Barcelona, Spain*

#*ICREA, Pg. Lluís Companys 23, Barcelona, 08010 Spain*

E-mail: german.salazar.alvarez@angstrom.uu.se

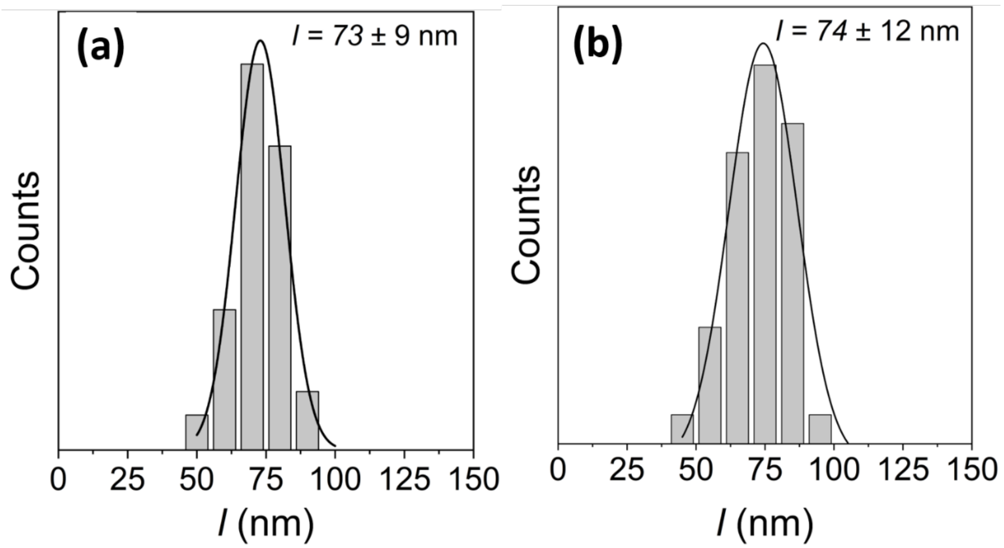

Figure S1: Log-normal size distributions of **(a)** pristine Fe_3O_4 and **(b)** $Li_xFe_3O_4$ (x = 1.5).

Size distributions of pristine ($x = 0$) and lithiated ($x = 1.5$) samples were obtained by manually measuring ca. 100 and ca. 30 nanocube edge lengths, respectively, using the image processing software *imageJ*.[1] The TEM images were calibrated for each magnification using the lattice parameter of a gold standard sample.

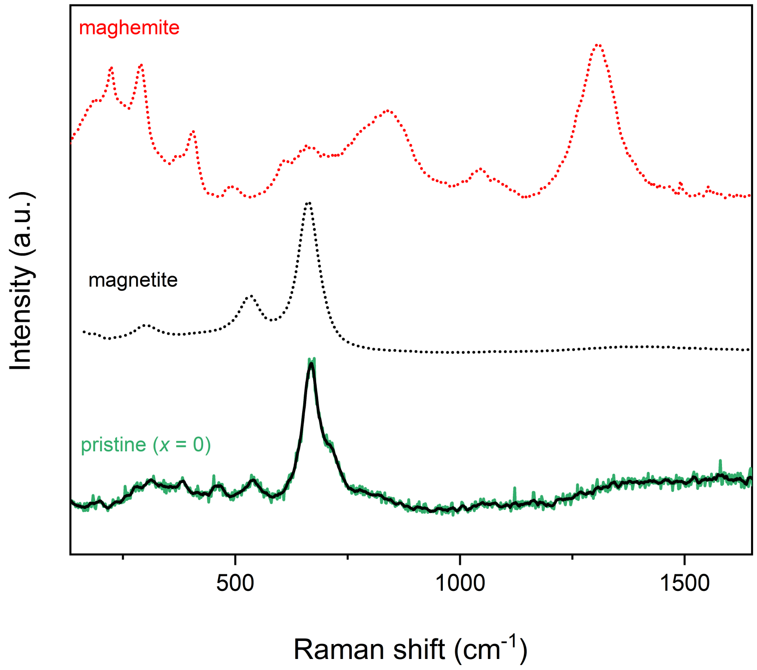

Figure S2: Raman spectrum of the pristine sample is presented together with those of magnetite (Fe_3O_4) and maghemite ($\gamma-Fe_2O_3$) phases obtained from the AMS-RRUFF database for $\lambda = 532$ nm. The corresponding RRUFF IDs for each phase is R060656 and R140712, respectively.[2] The extra peaks in the spectrum of pristine sample ($x = 0$) can be assigned to maghemite phase, which is possibly located at the surface layer of the particle.

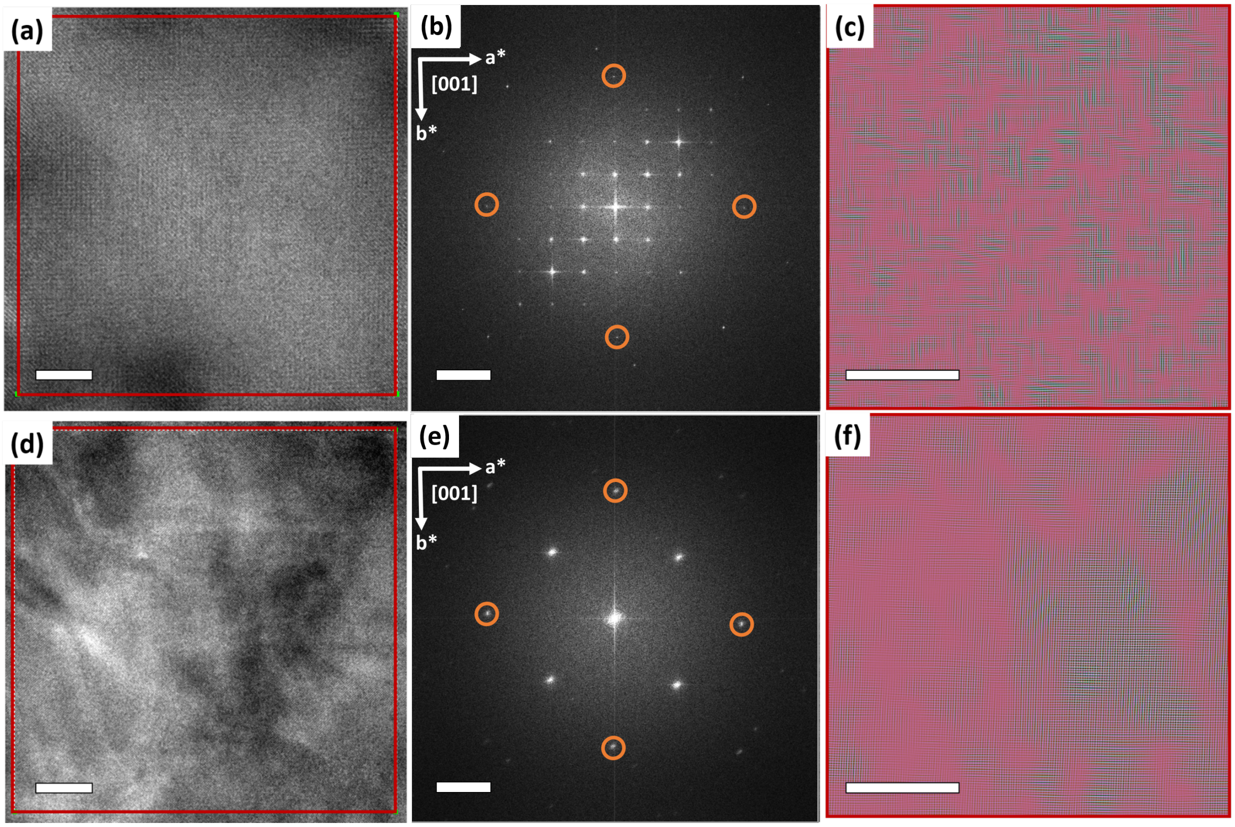

Figure S3: **(a, d)** HRTEM images of pristine ($x = 0$) and lithiated ($x = 1.5$) nanocubes (scale bar = 10 nm), with corresponding fast Fourier transform (FFT) analysis shown in **(b, e)** (scale bar = 2 nm^{-1}) and inverse FFT of $(400)_{sp}/(200)_{rs}$ reflections (indicated by circles in **(b, e)**) shown in **(c, f)** (scale bar = 10 nm)

.

Figure S3 (a) and (d) shows electron microscopy images of pristine ($x = 0$) and lithiated ($x = 1.5$) particles, respectively. The red square areas in (a) and (d) are selected for fast Fourier transformation (FFT) to obtain inter-atomic plane reflections shown in (b) and (e). The inverse FFT (iFFT) function is applied on chosen (400) reflections in (b) and (e)(denoted in circles). The obtained iFFT pattern from pristine sample ($x = 0$) in (c) shows monotonic contrast variation, whereas the lithiated sample ($x = 1.5$) in (f) demonstrates non-monotonic contrast variation rising from dislocations induced by lithiation.

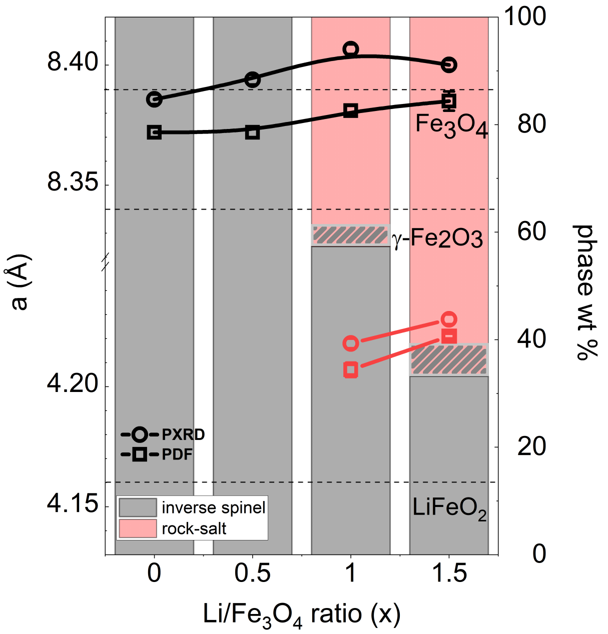

Figure S4: Histogram of volume weighted phase fractions from both Rietveld and PDFGui refinements (right-hand scale). The extra dash grey column represents difference in calculated phase fraction from PDF fit result. The obtained unit cell parameters from both Rietveld (PXRD) (circles) and PDFGui fits (squares) for each degree of chemical lithiation ($x = 0, 0.5, 1, 1.5$) marked in black and red to distinguish the spinel and rock-salt phases, respectively (left-hand scale). The horizontal dashed lines indicate lattice parameter values from literature for bulk Fe_3O_4, $\gamma-Fe_2O_3$, and $LiFeO_2$ phases.[3,4]

Figure S4 displays the calculated phase weight percentages and lattice parameters from Rietveld and PDFGui refinements. The overall trend by lithiation in both techniques has shown similar results; such as increase in the rock salt phase contribution and lattice parameter expansion of both spinel and rock salt phases.

Table S1: Structural information from PXRD analysis of $Li_xFe_3O_4$ (x = 0, 0.5, 1, 1.5) samples.

**Sample ID**			$x = 0$	$x = 0.5$	$x = 1$	$x = 1.5$
Spinel *Fd-3m* (*wt*%)			100	100	57	33
Rock salt *Fm-3m* (*wt*%)			0	0	43	67
Fd-3m	a (Å)		8.3857(9)	8.3939(8)	8.4066(9)	8.400(1)
	Fe1 (8a) occupancy		1.000(8)	1.000(7)	0.92(3)	0.67(3)
	Fe2 (16d) occupancy		0.938(7)	0.957(8)	1.00(3)	0.99(4)
	O (x)		0.2543(4)	0.2535(4)	0.2558(4)	0.2591(7)
	Bond lengths (Å)	Fe1-O	1.878	1.868	1.906	1.957
		Fe2-O	2.061	2.070	2.053	2.023
Fm-3m	a (Å)		-	-	4.2179(5)	4.2279(6)
	Li1 occ		-	-	0.34(2)	0.308(9)
	Fe2 occ		-	-	0.66(2)	0.69(1)
	Bond length (Å)	Li1/Fe2-O	-	-	2.109	2.114
Refinement params.	R_{wp}		1.089	1.062	1.707	1.389
	GOF		1.826	1.726	3.000	2.434

Table S2: Full width at half maximum ($FWHM$ (°)) obtained for the refined structures in $Li_xFe_3O_4$ (x = 0, 0.5, 1, 1.5) samples.

Fd-3m	$x = 0$	$x = 0.5$	$x = 1$	$x = 1.5$
111	0.375(3)	0.359(4)	0.362(3)	0.360(5)
220	0.396(4)	0.387(4)	0.385(3)	0.362(7)
311	0.389(5)	0.389(2)	0.388(2)	0.369(1)
400	0.480(4)	0.466(4)	0.427(2)	0.427(6)
333	0.443(3)	0.427(4)	0.418(6)	0.409(5)
440	0.443(3)	0.436(3)	0.430 (2)	0.421(4)
Fm-3m				
111	-	-	0.423(4)	0.401(4)
200	-	-	0.460(3)	0.435(3)
220	-	-	0.378(2)	0.362(3)
222	-	-	0.493(4)	0.460(3)

Table S3: Structural phase information from PDF analysis of $Li_xFe_3O_4$ (x = 0, 0.5, 1, 1.5) samples.

**Sample ID**			$x = 0$	$x = 0.5$	$x = 1$	$x = 1.5$
Spinel *Fd-3m* (*wt%*)			1	1	0.62(4)	0.400(3)
Rock salt *Fm-3m* (*wt%*)			0	0	0.37(4)	0.599(3)
Fd-3m	*a* (Å)		8.372(2)	8.372(2)	8.381(2)	8.385(4)
	Fe1 (8*a*) occupancy		1	1	1	1
	Fe2 (16*d*) occupancy		1	1	1	0.75
	O (x)		0.2546(7)	0.255(1)	0.255(1)	0.257(2)
	Bond lengths (Å)	Fe1-O	1.879	1.887	1.883	1.915
		Fe2-O	2.055	2.051	2.056	2.04
Fm-3m	*a* (Å)		-	-	4.207(3)	4.221(1)
	Bond lengths (Å)	Fe1-O	-	-	2.104	2.11
		Fe-Li	-	-	2.975	2.984
		Fe2-O	-	-	3.644	3.656
Refinement param.	R_w		0.12	0.12	0.09	0.10

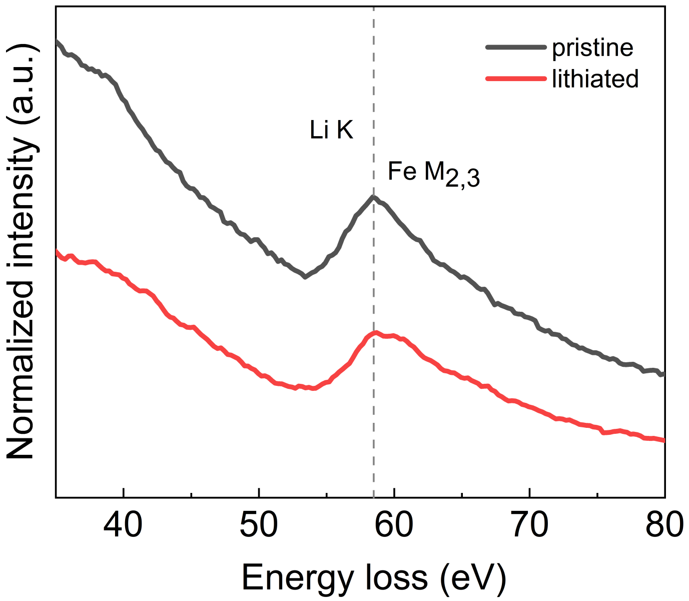

Figure S5: Electron energy loss spectra showing Li K-edge ($\sim$ 55 eV) and Fe $M_{2,3}$-edge ($\sim$ 54 eV) for pristine Fe_3O_4 and lithiated $Li_xFe_3O_4$ (x=1.5) samples. The dashed line indicates the Fe $M_{2,3}$-edge of the pristine sample for comparison.

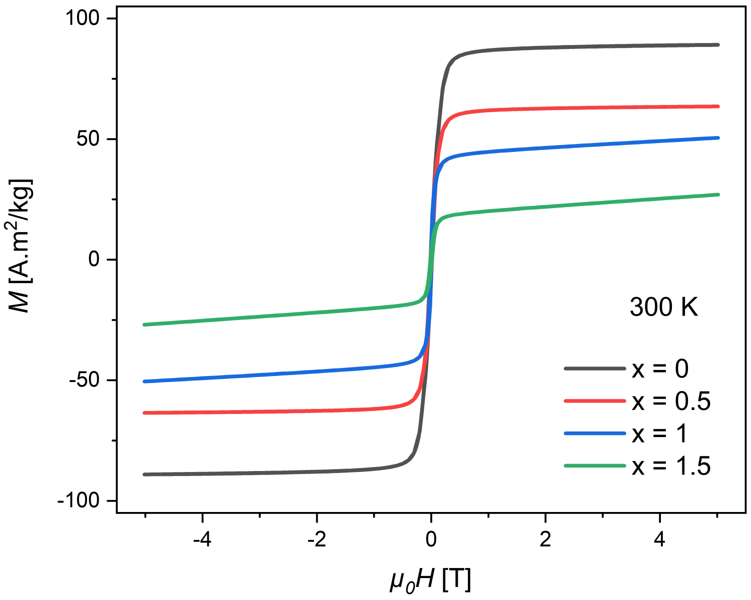

Figure S6: Hysteresis loops for the $Li_xFe_3O_4$ ($x = 0$, 0.5, 1, 1.5) samples recorded at $T = 300$ K.

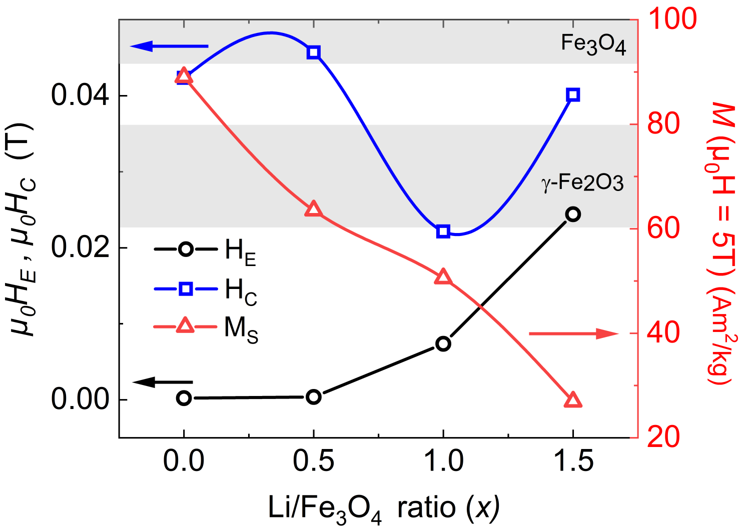

Figure S7: Plot of the dependence of the coercivity (H_C) and the exchange bias (H_E) at 10 K, after field-cooling, for $Li_xFe_3O_4$, $x = 0, 0.5, 1, 1.5$ (left axis). Magnetization values close to saturation ($M(\mu_0 H = 5$ T)) for each sample at an applied field of 5 T at 300 K (right axis). The shaded regions show the expected saturation magnetization values for bulk Fe_3O_4 and $\gamma{-}Fe_2O_3$ phases,[3] for comparison. Lines connecting the data points are guides to the eyes.

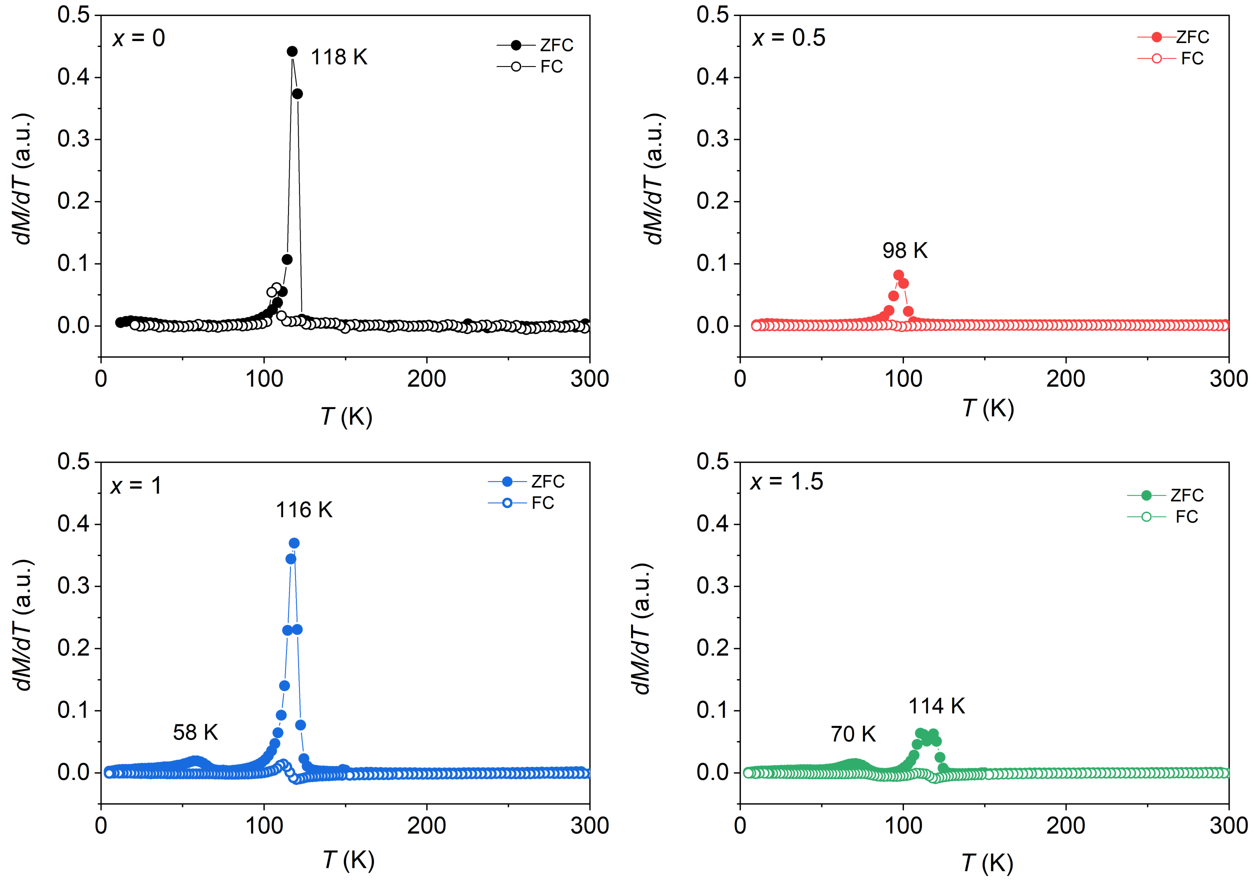

Figure S8: The derivative of ZFC-FC temperature dependent magnetization curves as a function of temperature for each $Li_xFe_3O_4$, $x = 0, 0.5, 1, 1.5$ sample.

References

(1) Schneider, C. A.; Rasband, W. S.; Eliceiri, K. W. NIH Image to Imagej: 25 Years of Image Analysis. *Nat. Methods* **2012**, *9*, 671–675.

(2) De Faria, D. L.; Venâncio Silva, S.; De Oliveira, M. Raman Microspectroscopy of Some Iron Oxides and Oxyhydroxides. *J. Raman Spectrosc.* **1997**, *28*, 873–878.

(3) Schwertmann, U.; Cornell, R. M. *Iron Oxides in the Laboratory*; John Wiley & Sons, Ltd, 2000; Chapter 3, pp 27–54.

(4) Cox, D. E.; Shirane, G.; Flinn, P. A.; Ruby, S. L.; Takei, W. J. Neutron Diffraction and Mössbauer Study of Ordered and Disordered $LiFeO_2$. *Phys. Rev.* **1963**, *132*, 1547–1553.
